# Suicide Attempt by Intravenous Potassium Self-Poisoning: A Case Report

**DOI:** 10.1155/2012/323818

**Published:** 2012-01-15

**Authors:** Florent Battefort, Emilie Dehours, Baptiste Vallé, Ahmed Hamdaoui, Vincent Bounes, Jean-Louis Ducassé

**Affiliations:** ^1^Pôle de Médecine d'Urgences, Centre Hospitalier Universitaire de Toulouse, Place du Docteur Baylac, SAMU 31, 31059 Toulouse Cedex 9, France; ^2^Pôle de Médecine d'Urgences, Centre Anti-Poison, SAMU 31, 31059 Toulouse, France

## Abstract

*Introduction*. Overdose of potassium is not as frequently encountered in clinical practice as hyperkalaemia due to acute or chronic renal disease. However, potassium overdoses leading to serious consequences do occur. *Case Presentation*. A 20-year-old nurse student presented with a cardiac arrest with asystole rhythm. Beside the patient were found four 50-mL syringes and empty vials of potassium chloride (20 mL, 10%). After initial resuscitation with epinephrine, 125 mL of a 4.2% intravenous solution of sodium bicarbonate were injected which resulted in the recovery of an effective cardiac activity. The patient recovered without sequelae. *Conclusion*. The difficulty in this case was to recognize the potassium poisoning. The advanced resuscitation with the use of a specific treatment helped to resuscitate the patient.

## 1. Introduction

Potassium chloride is extensively used as a potassium supplement, both by physicians as a therapeutic modality and by the general public, mostly in the form of salt substitute. Therapeutically, both oral and intravenous forms of potassium are used.

We describe a case of suicide attempt by intravenous injection of potassium, which led to heart attack, supported by a medical team of prehospital resuscitation.

## 2. Case Presentation

**Figure 1:**
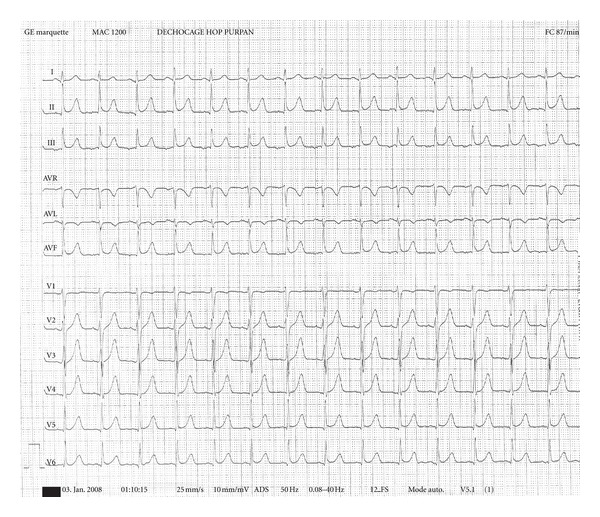
ECG shows sinus, regular rhythm, normal axis, and fine QRS, but there was a broad and sharp T wave.

The out-of-hospital medical emergencies service received a call from a person whose daughter, a 20-year-old nurse student, presented with a dyspnea with cyanosis and consciousness disorders. The mobile emergency and resuscitation service (SMUR) and firemen were sent on site. When firemen arrived, the patient was in cardiac arrest for less than 5 minutes according to witnesses. Cardiopulmonary resuscitation was immediately started associated with the implementation of a semiautomatic defibrillator without indicated shock. When SMUR arrived, the patient was in asystole. Besides the patient were found four 50-mL syringes and empty vials of potassium chloride (20 mL, 10%). The patient received an orotracheal intubation and mechanical ventilation as well as the implementation of peripheral venous access device with a 0.9% NaCl solution. A cutaneous injection site was found in the skin fold of the left elbow of the victim. Through this access, was injected an intravenous bolus of 1 mg of epinephrine and 125 mL of a 4.2% intravenous solution of sodium bicarbonate. After 20 minutes, initial management allowed getting a spontaneous circulation. Blood pressure was 160/100 mmHg, pulse of 130/min, and the O_2_ saturation to 99% with FIO_2_ of 1, EtCO_2_ was measured at 27 mmHg. The electrocardiogram showed sinus, regular rhythm, normal axis, and fine QRS, but there was a broad and sharp T wave (Figure 1).

The patient was secondarily sedated when signs of waking appeared, and she was transferred to intensive care unit with establishment of therapeutic hypothermia. In the end, the hemodynamic status was stable with a blood pressure of 135/88 mmHg and a pulse of 90/min. After one hour, biological tests found a natremia of 138 mmol/L, kalemia of 3.9 mmol/L and chloremia of 108 mmol/L. There was no renal failure (urea 2.5 mmol/L, creatinemia 78 *μ*mol/L). There was a hyperlactatemia at 4.5 mmol/L without acidosis (pH 7.36, pCO_2_ 35 mmHg, PO_2_ 150 mmHg, and HCO_3_ 20 mmol/L). The electrocardiogram performed at that time was unchanged.

The patient was extubated on day 1. After an initial period of delirium, she recovered a normal neurological status. Psychiatric hospitalization was decided in the aftermath of resuscitation.

## 3. Discussion

The originality of this case lies in the favourable development of this rare but often lethal [1–4] poisoning due to the introduction of an appropriate emergency therapy.

Potassium allows the depolarization of the myocardial cell and the cell of the sinoatrial node (concentration between 3.7 and 5.3 mmol/L [5]). Hyperkalemia will disrupt depolarization and repolarization of the myocardial cell as well as the good working of the sinoatrial node with ECG appearances, depending on kalemia. An ECG is then essential to detect and follow these abnormalities. There is an approximate relationship between plasmatic potassium concentration and electrocardiographic signs but with great variability [6]. In this case, the patient is in asystole during the supporting and preliminary rhythm disorders could not be detected. On the other hand, during the recovery of an effective cardiac activity, ECG shows broad, positive, sharp T waves as described in the literature.

Rapid diagnosis of intravenous potassium poisoning complicated by cardiac arrest helped in the implementation of a specific treatment of administration of sodium bicarbonate at a dose of 1 mmol/kg because of its effect on hyperkalemia by intracellular potassium transfer in 15 minutes and its duration of action for several hours [6]. The hypokalemic effect of sodium bicarbonate is more pronounced for persons previously in metabolic acidosis and conversely less marked among people with advanced renal failure or without metabolic acidosis. This loading dose in prehospital probably allowed to have a normal kalemia on admission of the patient in the resuscitation unit and to correct acidosis due to cardiac arrest. However, it would have been more efficient to use calcium chloride or calcium gluconate for which the onset of action is 1 to 3 minutes with a duration of action of 30 to 60 minutes [6]. It directly antagonizes the membrane effects of hyperkalemia. Calcium gluconate was not part of the armamentarium of the physician.

Other medications may be given in an emergency such as beta-2-adrenergic agonists. Their use remains controversial because of the risk of tachycardia and coronary failure for people at risk [6]. The administration of insulin associated with 30% glucose-added serum is also known for treatment of hyperkalemia by stimulating cellular uptake of potassium. It allows a decrease of plasmatic potassium concentration from 0.5 to 1.5 mmol/L in 15 minutes with a peak at 60 minutes and for several hours [6]. The ion exchange resins (sodium polystyrene sulphonate) and diuretics have no place in this type of care. Their administration and their delayed effect dedicate them to the treatment of chronic moderate hyperkalemia. Hemodialysis performed in emergency is the standard treatment of hyperkalemia, particularly in cases of severe hyperkalemia which quickly set up or with high risk of reconstitution (hemolysis, tissue catabolism) [6]. Its use was not recommended in our case since the intravenous therapy of sodium bicarbonate conducted to a normal kalemia.

Considering the case presentation and the administration mode, the bolus effect of potassium caused the cardiac arrest, but the total injected dose of potassium was not important. This explains why a single intravenous sodium bicarbonate treatment leads back to a normal kalemia on arrival at hospital.

This mode of poisoning is rare, it occurs only in cases of specific patients such as those including medical or paramedical personnel. We did not find any similar cases described in the literature. Most toxic hyperkalemia described are due to drug intakes [7, 8], side effects, or overdose of medical treatments [9].

## 4. Conclusion

Intravenous potassium intoxication is a rare poisoning. We must learn to mention it when patient are working in the medical community. The difficulty in this case is to recognize the potassium poisoning. On the other hand, the treatments themselves are coded, prioritized, and accessible from the prehospital phase. Advanced resuscitation with the use of a specific treatment by sodium bicarbonate helped to resuscitate the patient.
